# The molecular mechanisms and physiological roles of mitochondria dynamics in *Saccharomyces cerevisiae*

**DOI:** 10.15698/mic2025.08.859

**Published:** 2025-08-27

**Authors:** Chang-Lin Chen, Wei-Ling Huang, Alexander Rapoport, Rimantas Daugelavičius, Chuang-Rung Chang

**Affiliations:** 1 Institute of Biotechnology, National Tsing Hua University, 101, Section 2, Kuang-Fu Rd., Hsinchu City, 300044, Taiwan.; 2 Institute of Microbiology and Biotechnology, Faculty of Medicine and Life Sciences, University of Latvia, Jelgavas Str., 1-537, Riga, LV-1004, Latvia.; 3 Research Institute of Natural and Technological Sciences, Vytautas Magnus University, K. Donelaičio str. 58, 44248, Kaunas, Lithuania.; a Current address: Facility Division, National Synchrotron Radiation Research Center, 101, Hsin-Ann Road, Hsinchu City, 300092, Taiwan.

**Keywords:** mitochondria, yeast, dynamic organelles

## Abstract

Mitochondria are essential organelles that form a dynamic network within cells. The fusion, fission, and transport processes among mitochondria must reach a balance, which is achieved through complex regulatory mechanisms. These dynamic processes and regulatory pathways are highly conserved across species and are coordinated to help cells respond to environmental stress. The budding yeast *Saccharomyces cerevisiae* has become an important model organism for studying mitochondria dynamics due to its genetic tractability and the conservation of key mitochondrial regulators. Previous research on mitochondria dynamics in yeast has provided valuable insights into the regulatory pathways in eukaryotic cells. It has helped to elucidate the mechanisms related to diseases associated with disrupted mitochondria dynamics. This review explores the molecular mechanisms underlying mitochondria dynamics and their physiological roles in *Saccharomyces cerevisiae*. The knowledge we learned from the primary eukaryotic yeast cell will aid us in advancing future research on the regulatory mechanisms of mitochondria in both health and disease.

## Abbreviations

CJ - cristae junction,

ER - endoplasmic reticulum,

ERMES - ER-mitochondria encounter structure,

MDC - mitochondria-derived compartment,

MDV - mitochondria-derived vesicle,

MICOS - mitochondrial contact site and cristae organizing system,

mtDNA - mitochondrial DNA.

## INTRODUCTION

Endosymbiotic mitochondria maintain double-membrane features and circular genomes in eukaryotes 1. Mitochondria are involved in almost all essential metabolic activities, including fatty acid oxidation, iron-sulfur cluster biogenesis, energy transformation, calcium homeostasis, and apoptosis in eukaryotic cells 2. These semiautonomous organelles have their own transcription and translation systems 3. The mitochondrial genome (mtDNA) encodes genes required for the assembly of oxidative phosphorylation complexes. Mitochondrial translation coordinates with the cytosolic translation system to synthesize oxidative phosphorylation complexes to meet cellular energy demands 3. Recently, new roles of mitochondria have been vigorously explored in different model organisms. For example, mitochondria serve as proteasomes that accumulate unfolded proteins for protein degradation 4. Additionally, mammalian mtDNA nucleoids can be extruded into the cytoplasm and activate immune responses 5,6. These reports indicate that mitochondria serve not only as cellular powerhouses but also as hubs for pivotal metabolism.

Microscopy with mitochondria-targeting dyes and fluorescent proteins has demonstrated the dynamic alternations of the organellar network in real-time in yeast and mammalian cells. These morphological changes can be attributed to two major processes: fission and fusion 7. Proper regulation of the dynamic mitochondrial network is crucial for maintaining organellar quantity and quality. The fission process facilitates mitochondrial trafficking and inheritance. Additionally, damaged and depolarized mitochondria can be sequestered for degradation after fission. Conversely, the fusion process promotes the exchange of components to reduce detrimental accumulation in individual mitochondria 8. Mitochondria dynamics regulate mitochondrial quality and organellar communication 9. Abnormal mitochondrial morphologies were found in degenerative neurons, premature aging, and tumor cells 10. The ability to shift the balance of mitochondria dynamics has become a critical index for evaluating the health of mitochondria. Genetic manipulation to restore the balance of disrupted mitochondria dynamics was demonstrated to rescue disease-associated phenotypes 11. Hence, the regulatory factors of mitochondria dynamics have been scrutinized recently. Mitochondrial dynamic factors are classified as either fusion or fission factors. Additional proteins were discovered to facilitate the shaping of the mitochondrial network. These factors have been shown to regulate mitochondrial fission and fusion through either specific protein-protein interactions or unique enzymatic machinery. However, evidence has indicated that unidentified factors, which may participate in redundant pathways or become active under particular conditions, remain to be characterized.

**Figure 1:**
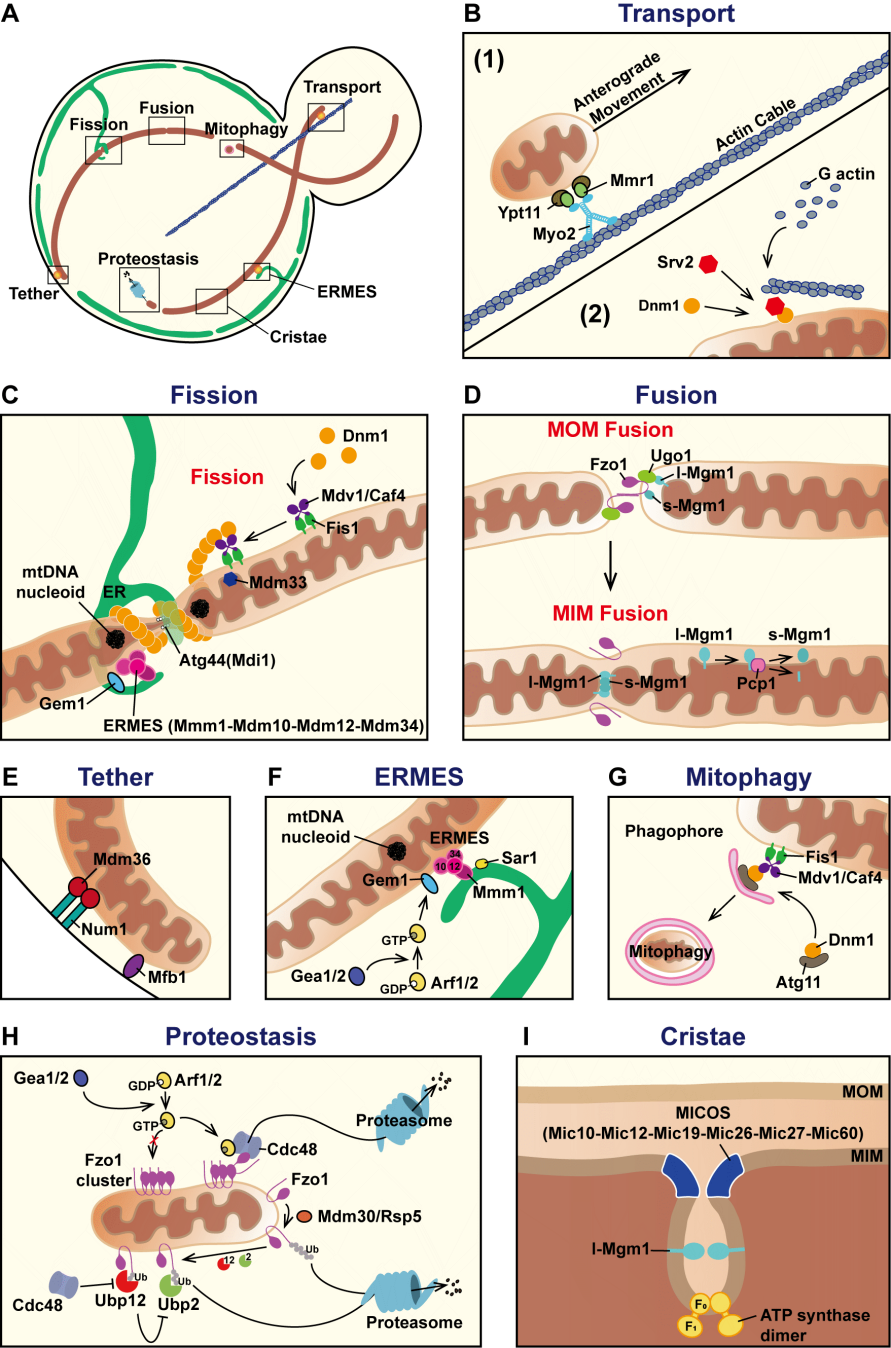
FIGURE 1: Scheme of the factors regulating mitochondria dynamics in yeast cells. **(A)** Fission, fusion, and transport processes regulate mitochondria dynamics in yeast. This cartoon provides an overview of the regulators that participate, including plasma membrane tethers, ER-mitochondria encounter structure (ERMES), mitophagy, proteostasis, and cristae organization. Indicated boxes correspond to separate panels **(B-I)**, each illustrating the detailed molecular mechanisms of the indicated dynamic process. **(B)** Transport: (1) The Myo2-Mmr1-Ypt11 complex carries mitochondria by connecting them along with actin cables to facilitate anterograde movement through the bud neck of the yeast cell. (2) Srv2 interacts with Dnm1 on mitochondria to promote fission. **(C)** Fission: Dnm1 is recruited from the cytosol to the mitochondrial surface by its receptor Fis1, with adaptors Mdv1 and Caf4 facilitating its recruitment. At the fission site, Dnm1 assembles into a spiral-ring structure to constrict the outer membrane. Atg44/Mdi1 in the intermembrane space generates inner membrane curvature to complete the fission process. The ER and ERMES, which localize at fission sites, are meant to promote fission. mtDNA nucleoids are segregated during fission. **(D)** Fusion: Mitochondrial outer membrane (MOM) is initiated by docking via Fzo1, which promotes the closing and further fusion of two mitochondria. Fzo1 at MOM and Mgm1 at mitochondrial inner membrane (MIM) are linked by Ugo1 to coordinate subsequent MIM fusion. Mgm1 isoforms are processed by the mitochondrial protease Pcp1. **(E)** Tether: Num1 and Mfb1 act as independent plasma membrane anchors that link mitochondria to the cell cortex, maintaining proper mitochondrial distribution. **(F)** ERMES: The ER-mitochondria encounter structure is composed of Mmm1, Mdm10, Mdm12, and Mdm34, which localize at contact sites of ER (connected by Mmm1) and mitochondria (connected by Mdm10-Mdm12-Mdm34). ERMES marks the potential fission sites and contributes to mtDNA maintenance. Gem1, regulated by Arf1/2 and Gea1/2, regulates ERMES size and number. Sar1 is an ER protein that promotes membrane curvature and lipid exchange between the ER and mitochondria to maintain mitochondrial morphology. **(G)** Mitophagy: Damaged mitochondria are selectively removed by the mitochondrial fission process. Dnm1 interacts with Atg11, a scaffold protein at the phagophore assembly site, to support mitochondrial clearance. **(H) **Proteostasis: Fzo1 turnover is controlled through proteolytic and ubiquitin-dependent pathways. During log phase, the deubiquitylases Ubp12 and Ubp2 modulate the Fzo1 level by controlling Fzo1 ubiquitinylation status, and Cdc48 promotes the degradation of Ubp12, thus stabilizing Ubp2 and facilitating fusion. In Arf1/2 mutants, Fzo1 forms clusters that hinder fusion, which are removed by Cdc48 overexpression. Under vacuolar stress, Fzo1 is targeted for ubiquitination by the E3 ubiquitin ligases SCF^Mdm30^ and Rsp5, causing fusion inhibition. **(I)** Cristae: The ultrastructure of MIM is shaped by three regulators, the mitochondrial contact site and cristae organizing system (MICOS), Mgm1, and F_1_F_0_-ATP synthase dimers. MICOS localizes at the cristae junction between inner boundary membrane and cristae membrane. Mgm1 mostly localizes at flat lamellar region of cristae. F_1_F_0_-ATP synthase dimers assembles in rows to generate curvature at cristae tips.

The budding yeast *Saccharomyces cerevisiae* is a powerful model for easy genetic manipulation, and its genome is highly conserved compared to that of higher eukaryotes such as mammals. Additionally, budding yeast has a short cell cycle to proliferate, making them helpful in establishing replicative or chronological senescence research 12. Scientists have utilized the budding yeast as a model organism to elucidate the underlying molecular mechanisms of mitochondria dynamics (**Figure 1**). One unique advantage of this primary eukaryotic model is that yeast can survive under respiratory growth defects, i.e., the *rho^0^* strain. This phenomenon is a powerful feature of the acquisition of mitochondrial dysfunction-related phenotypes. Furthermore, yeast has been utilized to model numerous human mitochondrial diseases, including Parkinson’s disease 13,14, Barth syndrome 15,16, Leigh syndrome 17,18, and Friedreich’s Ataxia 19,20. These models enable functional validation of disease-associated gene mutations and facilitate preliminary drug or compound screening for therapeutic development. The unique shape of budding yeast cells makes them an effective platform for examining mitochondrial network morphology and distribution 21. These features provide advantages for characterizing the regulatory mechanisms of mitochondria dynamics in yeast. To date, abundant evidence has shown that mitochondrial network morphology is present in distinct types caused by deletions of specific genes. To better understand mitochondria dynamics in yeast cells, we reviewed the literature on the dynamic mechanisms and physiological roles of mitochondria in budding yeast.

## LARGE GTPases CONSTITUTE THE PROMINENT PROTEIN FAMILY THAT MEDIATES MITOCHONDRIA DYNAMICS

**Figure 2:**
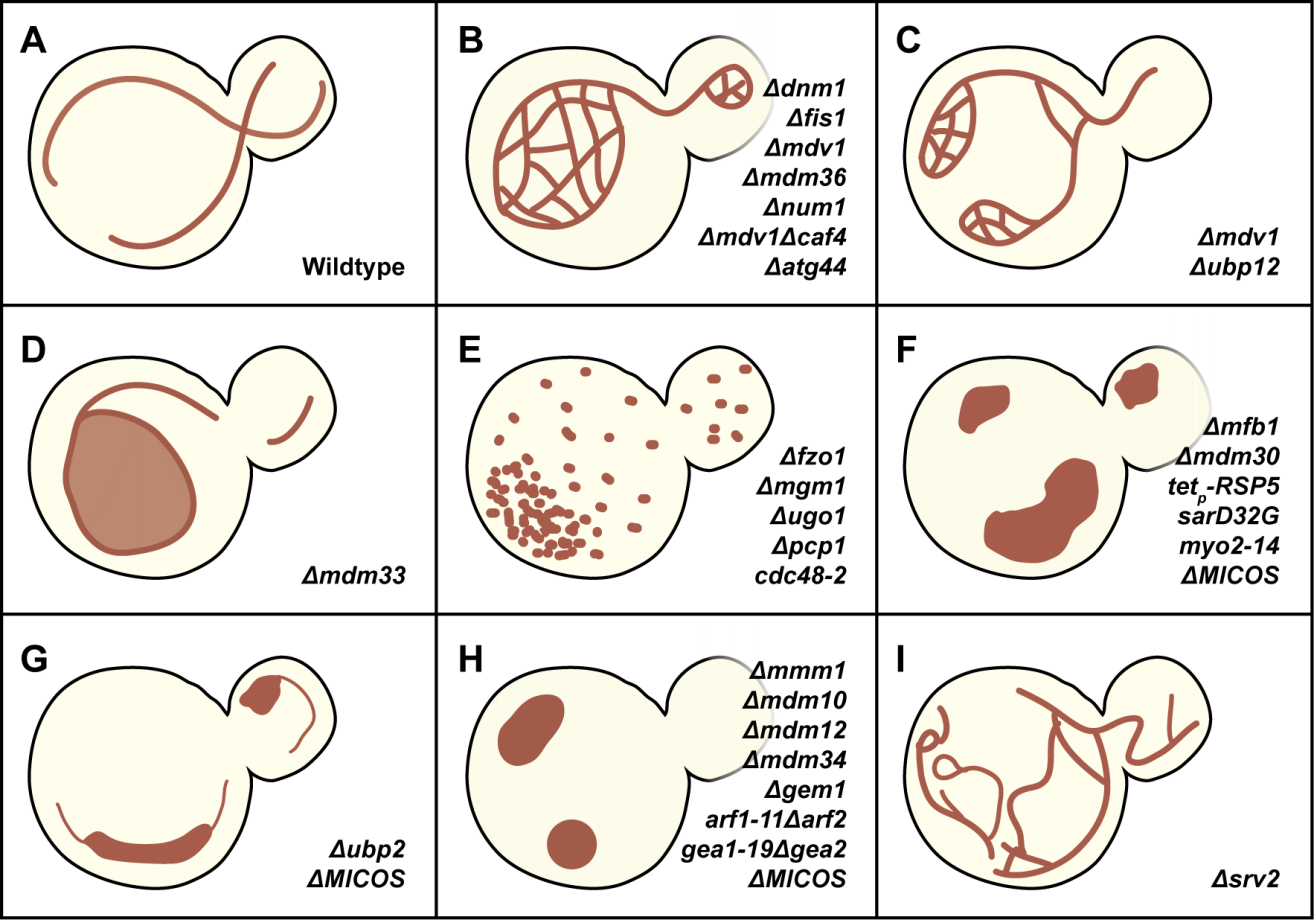
FIGURE 2: Illustration of mitochondrial morphology phenotypes in yeast cells. Representative mitochondrial morphology phenotypes are demonstrated in each panel, which are identified in the indicated genetic ablation or mutation strains. **(A)** In vegetative wild-type yeast, mitochondria present a long tubular network along the cell cortex. **(B-C)** Fission defects give rise to a mesh net **(B)** or collapsed net **(C)** morphology. **(D)** Disintegrated lipid homeostasis of mitochondria induces a lariat-like network. **(E)** Fusion defects result in fragmented mitochondria. **(F-H)** Loss of function in inter-organellar contacts with mitochondria causes abnormal distribution of big puncta mitochondria **(F-G)** or globular **(H)** mitochondrial morphology. **(I)** Loss of actin organization caused hyperfusion of mitochondria.

Mitochondrial networks continuously reorganize in eukaryotic cells 22. Mitochondria dynamics are executed mainly by fission and fusion processes 2. In vegetative growing yeast cells, mitochondria are present in long tubules at the cell cortex, that have few branches (**Figure 2A**). We can monitor frequent mitochondrial fission and fusion events via real-time microscopy by expressing mitochondrial-targeting fluorescent proteins. These dynamic processes are conducted by a group of large dynamin-related GTPase proteins, such as Dnm1 and Fzo1 in yeast (Drp1 and Mfn1/2 in mammalian cells), which are often referred to as mitochondria dynamics factors 23,24,25. Accordingly, mitochondrial fission and fusion are accompanied by GTP hydrolysis, which is thought to alter the protein conformation of dynamic factors to facilitate fusion and fission 26. This phenomenon can be regulated by post-translational modifications in mammalian cells 27,28,29. The human orthologs of dynamic factors are listed in **Table 1**.

In yeast mitochondrial fission, the Dnm1-Fis1 axis is the primary regulatory pathway 30. Dnm1 is an 85-kDa cytosolic GTPase that participates in mitochondrial fission 23,25. It is recruited to constriction sites and assembles into a spiral/ring structure to excise the mitochondrion into two halves 31. Interestingly, only a subset of mitochondria-associated Dnm1 clusters undergo GTP hydrolysis to drive active fission 32. Dnm1 defects cause a hyperfused, mesh-net mitochondrial morphology with short tubules extending from the net (**Figure 2B, C**) 25. Fis1 is a mitochondrial outer membrane protein that serves as a receptor for Dnm1, regulating Dnm1 recruitment via physical interactions 33. Two adaptors, Mdv1 and Caf4, also participate in the fission process by interacting with Dnm1 and recruiting Dnm1 to the mitochondrial outer membrane alongside Fis1 32,34. Notably, Mdv1, but not Caf4, is required to activate membrane scission with Dnm1 33. Mesh-net mitochondria have also been found in Δ*fis1* and Δ*mdv1*Δ*caf4* cells 34. Recently, a small mitochondrial intermembrane space protein, Atg44/Mdi1, has been characterized as a fission factor. Although lacking a GTPase domain, Atg44 coordinates with Dnm1 to facilitate mitochondrial fission 35,36. Notably, Atg44 mediates mitophagy, a selective degradation of mitochondria by autophagy machinery, by executing mitochondrial fission independently of Dnm1. Moreover, *in vitro* analysis suggests that Atg44 accumulates near the lipid membranes, potentially promoting membrane bending and contributing to complete mitochondrial fission 37. Mitochondrial fission is primarily conducted through a stepwise mechanism, including coordinated interactions of Dnm1, Fis1, Mdv1, and Caf4, with additional modulation by Atg44 (**Figure 1C**). Beyond these core components, genetic screenings identified additional proteins aimed at manifesting mitochondrial fission pathways. One example is Mdm33, which regulates mitochondrial membrane lipid homeostasis to influence morphological changes. An increase in Dnm1-dependent fragmented mitochondria has been reported in *MDM33*-overexpressing cells 38. The Δ*mdm33* strain possesses lariat mitochondria (**Figure 2D**). Although this morphology is unlike the mesh-net morphology found in Δ*dnm1*, an elongated, irregular mitochondrial network makes Mdm33 a potential regulator of mitochondrial fission 39. Another example is Mdm36, an antagonist of mitochondrial fusion machinery. Loss of Mdm36 reduces the number of Dnm1 foci on mitochondria and decreases the ratio of mitochondria associated with the cell cortex 40. These phenotypes indicate that Mdm36 may also be a supportive adaptor for mitochondrial fission.

**Table 1:** Mitochondria dynamics players in *Saccharomyces cerevisiae*.

**Yeast Gene**	**Human Ortholog**	**Role and Function**	**Reference**
**Major factors involved in mitochondrial fission**			
*DNM1*	DRP1	Cytosolic GTPase regulates mitochondrial outer membrane fission.	23 25
*FIS1*	FIS1	Mitochondrial outer membrane GTPase regulates recruitment of Dnm1.	33
*MDV1*	-	GTPase on the surface of mitochondrial outer membrane interacts with Dnm1 and Fis1 to facilitate mitochondrial fission.	32
*CAF4*	-	GTPase on the surface of mitochondrial outer membrane stabilizes Dnm1-Fis1 interaction to facilitate mitochondrial fission.	34
*ATG44/MDI1*	-	Mitochondrial intermembrane space protein generates membrane fragility to complete membrane scission	35 36
**Major factors involved in mitochondrial fusion**			
*FZO1*	MFN1/2	Mitochondrial outer membrane GTPase regulates mitochondrial outer membrane fusion.	24 49
*MGM1*	OPA1	Mitochondrial inner membrane GTPase regulates mitochondrial inner membrane fusion and maintains lamellar cristae structure.	53 54
*UGO1*	SLC25A46 (functional 114)	Mitochondrial outer membrane GTPase interacts with Fzo1 and Mgm1 to promote mitochondrial fusion.	58 59
**Accessory mediators for mitochondrial fission and fusion**			
*MDM33*	CCDC51 (structural 115)	Mitochondrial inner membrane protein regulates mitochondrial inner membrane fission and lipid homeostasis.	38 39
*MDM36*	-	Mitochondrial outer membrane protein regulates Dnm1-Num1 interaction with mitochondria on cell cortex.	40
*NUM1*	-	Cell cortex protein regulates mitochondria anchor on cell cortex.	73
*MFB1*	-	Mitochondria-associated F-box protein regulates mitochondria anchor on cell cortex.	72 73
*MMR1*	-	Mitochondria-associated protein supports mitochondria-actin linker for proper transport.	116
*MYO2*	MYO5A/B/C	Mitochondria-associated protein supports mitochondria-actin linker for proper transport.	89 116
*MMM1*	-	ERMES core protein regulates mitochondrial fission and MDC formation.	47 79 80
*MDM10*	-	ERMES core protein regulates mitochondrial fission and MDC formation.	47 79 81
*MDM12*	-	ERMES core protein regulates mitochondrial fission and MDC formation.	47 79 82
*MDM34*	-	ERMES core protein regulates mitochondrial fission and MDC formation.	47 79 83 86
*GEM1*	RHOT (Miro)	ERMES associated protein regulates MDC formation.	47
*SAR1*	SAR1	Small GTPase on ER regulates the size of ER-mitochondria contacts and membrane curvature of mitochondria and ER.	93
*SRV2*	CAP	Actin-binding protein interacts with Dnm1 to regulate mitochondrial fission.	102 104
**Regulatory mediators in mitochondrial fission and fusion**			
*CDC48*	VCP	Cytosolic AAA-ATPase regulates ubiquitinylation of Fzo1.	51
*MDM30*	-	Cytosolic F-box protein regulates ubiquitinylation of Fzo1.	117 118
*UBP2, UBP12*	USP	Cytosolic deubiquitylase regulates ubiquitinylation of Fzo1.	51
*PCP1*	PARL	Mitochondrial inner membrane protease regulates proteolytic process of Mgm1.	55
*ARF1, ARF2*	ARF	Small GTPase on Golgi negatively regulates Fzo1 turnover.	91
*GEA1, GEA2*	GBF1	Guanine nucleotide-exchange factor of Arf1/2 negatively regulates Fzo1 turnover.	91
**Other factors related to mitochondria dynamics**			
*MIC10, MIC12, MIC19, MIC26, MIC27, MIC60*	Mic10, Mic12, Mic19, Mic26, Mic27, Mic60	MICOS subunits in mitochondrial inner membrane regulates mitochondrial inner membrane ultrastructure.	62 63 64
*ATP20, ATP21*	ATP5L, ATP5I	F_1_F_0_-ATP synthase subunit regulates dimerization and maintains membrane curvature of cristae.	65 66 67
*AAC2*	ANT1	Mitochondrial adenine nucleotide translocator regulates mitochondrial fusion.	71
*CLU1*	CLUH	Cytosolic protein regulates mitochondrial outer membrane fission.	70
*SYM1*	MPV17	Mitochondrial inner membrane protein regulates mitochondrial inner membrane ultrastructure.	75

Mitochondrial fission contributes to mitochondrial derivatives and organellar activities, including the formation of mitochondria-derived vesicles (MDVs), peroxisomes, and the engulfment of mitophagosomes 41,42,43. MDV was first reported by Heidi McBride’s group as a mechanism for transporting mitochondrial proteins to peroxisomes as part of a proteostasis pathway in mammals 44. In senescent yeast, a functionally equivalent protein transport pathway involves the production of mitochondrial-derived compartments (MDCs) for selectively sorting mitochondrial proteins 45. MDC generation requires Dnm1/Fis1 machinery or other specific factors to facilitate fission 46. Under rapamycin treatment, MDC formation is supported by Dnm1 and the endoplasmic reticulum-mitochondria encounter structure (ERMES) 45,47. Additionally, Dnm1 plays a role in mitophagy by interacting with the Atg11 scaffold protein to promote mitochondrial fission and further clearance of damaged mitochondria 48 (**Figure 1G**).

Concerning yeast mitochondrial fusion, the Fzo1-Mgm1-Ugo1 pathway has been well characterized. Fzo1 is a 97 kDa mitochondrial outer membrane protein. It is also known as yeast mitofusin, with a GTPase domain facing the cytoplasm 24,49. Fzo1 facilitates the docking of two proximal mitochondria and the subsequent fusion of their outer membranes (**Figure 1D**). However, *in vitro* assays have revealed that Fzo1 overexpression leads to the opposite effect, as fragmented mitochondria accumulate. This result is potentially explained by Fzo1 overoccupying the membrane, which may disrupt the interactions of lipid molecules necessary for mitochondrial fusion 50. The turnover of Fzo1 is regulated by the proteolytic mechanism rather than transcriptional control (**Figure 1H**). Overexpression of Cdc48, an AAA ATPase with protein-unfolding activity, promotes the removal of Fzo1 clusters. In the exponential growth phase (log phase), the Fzo1 level is controlled by two deubiquitinases, Ubp12 and Ubp2, both of which are regulated by Cdc48 51. Under senescence or vacuolar inhibition conditions, Fzo1 is regulated by E3 ubiquitin ligases SCF^Mdm30^ (Skp1-Cullin-F-box complex with Mdm30 as the F-box protein) and Rsp5 through a proteolytic cascade 52. Mgm1 is a 99 kDa GTPase localized at the mitochondrial inner membrane, particularly regulating inner membrane fusion 53 (**Figure 1D**). *In vitro* evidence has shown that Mgm1 promotes fusion by tethering mitochondrial membranes 54. *In vivo*, Mgm1 defects cause the aggregation and fragmentation of mitochondria 53. Mgm1 has two isoforms, l-Mgm1 and s-Mgm1, which are processed by the mitochondrial protease Pcp1 55. Mgm1 isoforms are crucial for maintaining the ultrastructure of the mitochondrial inner membrane, with l-Mgm1 enriched in the cristae membrane and s-Mgm1 in the inner boundary membrane 56. Intriguingly, impairing the proteasome can restore mitochondrial fusion activity without Fzo1 by increasing the ratio of s-Mgm1/l-Mgm1 57. Ugo1, a mitochondrial outer membrane protein, regulates the Fzo1-Mgm1 interaction to promote the fusion of the mitochondrial outer and inner membranes 58,59. Fzo1, Mgm1, and Ugo1 form the axis of fusion factors that regulate mitochondrial fusion. Losing one of these genes results in fragmented mitochondria (**Figure 2E**).

Cristae formation, which refers to the ultrastructure of the mitochondrial inner membrane, is tightly regulated by three key factors in budding yeast: the mitochondrial contact site and cristae organizing system (MICOS) complex, fusion machinery, and the dimerization of F_1_F_0_-ATP synthases 60 (**Figure 1I**). Typically, cristae present a lamellar morphology through invagination of inner membrane. However, defects in the aforementioned factors can lead to abnormal cristae structures, such as onion-like or swollen architectures, as validated by electron microscopy 61. The MICOS complex consists of six subunits: Mic10, Mic12, Mic19, Mic26, Mic27, and Mic60, and is localized at the cristae junction (CJ) between the inner boundary membrane and the crista membrane 62,63. The stabilization of CJs has been shown to preserve the inner membrane ultrastructure and respiration capability 63,64. Fusion factor Mgm1 is also involved in the formation of lamellar cristae. Immuno-electron microscopy results have revealed that the s-Mgm1 is predominantly localized at the intermembrane space, suggesting that s-Mgm1 facilitates cristae formation immediately following outer membrane fusion events 56,60. The bending region of the cristae membrane requires the dimerization of F_1_F_0_-ATP synthases. Loss of dimerization-associated subunits Atp20/Atp21 disrupts cristae morphology 65,66. Studies have demonstrated that dimerization of F_1_F_0_-ATP synthase is formed in rows to maintain the curvature of the inner mitochondrial membrane in yeast 67,68. A recent study has revealed Mmc1/Mug99, an inner mitochondrial membrane protein, in *Schizosaccharomyces pombe* (fission yeast) as a novel factor that facilitates cristae maintenance by interacting with Mic60 and Mic26. However, there is no identified homolog of Mmc1 in budding yeast or humans. It is noteworthy that the expression of *Sp*Mmc1 in *S. cerevisiae* lacking MICOS components was able to restore respiratory growth defects. However, it was unable to rescue the altered mitochondrial morphology resulting from disrupted cristae structure. 69. These findings demonstrate the coordinated regulation of MICOS, Mgm1, and F_1_F_0_-ATP synthase in maintaining cristae formation, which is critical for respiratory function and mtDNA integrity.

In addition to conventional dynamic factors that directly bind to the mitochondrial membrane, researchers have endeavored to identify novel factors involved in regulating mitochondria dynamics. Accumulating evidence has indicated alternative mechanisms that govern mitochondrial network morphology and distribution. In yeast, Clu1, a functional homolog of the *cluA* gene in *Dictyostelium discoideum*, has been shown to mediate mitochondrial morphology similarly to Dnm1. Knocking out of *CLU1* results in aggregated or hyperfused mitochondria without impairing cell growth and respiratory function 70. Another evidence pertains to *AAC2*, an adenine nucleotide translocator localized at the mitochondrial inner membrane. The repression of *AAC2* causes mitochondrial fragmentation, even though *AAC2* has been confirmed to be dispensable for mitochondrial fusion 71. The underlying mechanism by which Clu1 or Aac2 operates remains to be explored. Several linker proteins have been proposed at the cell cortex to stabilize contacts between mitochondria and the cortical membrane. Num1 and Mfb1 independently mediate mitochondrial anchoring in the mother cell (**Figure 1E**), while Mmr1 interacts with Myo2 to transport mitochondria into the daughter cell 72,73,74 (**Figure 1B**). Genetic disruption of these anchors causes abnormal mitochondrial distribution and partial collapse of the mitochondrial network (**Figure 2F**). Sym1, identified as the yeast homolog of the human MPV17, which is mutated in a mitochondrial DNA depletion syndrome, also contributes to inner membrane integrity. The deletion of *SYM1* caused annihilated cristae structure, defective succinate dehydrogenase, and reduced respiratory activity 75.

An inability to change the balance of mitochondria dynamics would aid pathogenesis in mammalian cells. Thus, characterizing the dynamics factor in yeast cells is critical for all cells. Large GTPases work alongside other factors through protein-protein interactions and GTP hydrolysis to maintain constant mitochondrial fission and fusion. Dynamic processes are important for maintaining the quantity and quality of mitochondria. **Table 1** lists the abovementioned factors.

## THE ENDOPLASMIC RETICULUM PLAYS A CRITICAL ROLE IN MITOCHONDRIAL FISSION

Interorganellar contacts facilitate the exchange of lipids between mitochondria and other membrane-bound organelles, such as the endoplasmic reticulum (ER) and vacuoles, to create a dynamic circulating system 76. Many reports have demonstrated that ER-mitochondria contacts modulate mitochondrial division, lipid transfer, and ion homeostasis 77. In budding yeast, these contacts are regulated by ERMES, which forms a bridge-like structure, as revealed by cryo-correlative light and electron microscopy (cryo-CLEM) 78 (**Figure 1F**). ERMES consists of four core proteins: Mmm1, Mdm10, Mdm12, and Mdm34 79. The genetic disruption of any of these four genes results in abnormal spherical mitochondrial morphology and impaired mitochondrial inheritance 80,81,82,83 (**Figure 2H**). Based on these findings, ERMES is considered to play a critical role in mitochondria dynamics. Jodi Nunnari's group was the first to report how ER contributes to mitochondrial fission in yeast and mammalian cells. Their study demonstrated ER tubules wrap around mitochondria and mark the mitochondrial fission site 84 (**Figure 1C**). The fission factor Dnm1 assembles into clusters at these sites and executes mitochondrial division. ERMES foci were later recognized as ER-associated mitochondrial fission sites. Recent research has revealed that ER tubules and Dnm1 denote fission and fusion sites on mitochondria 85. Since mtDNA nucleoid segregation has been demonstrated to be associated with mitochondrial division, studies have indicated that ERMES colocalizes with mtDNA nucleoid spots and contributes to mtDNA maintenance 82,83,86,87,88. Moreover, the colocalization of Mdm10 and Mdm12 with mtDNA nucleoids occurs independently of actin filaments 87. The spherical mitochondrial network morphology phenotype in ERMES-defective cells is unrelated to Myo2-mediated, actin-associated anterograde mitochondrial transport 89.

Studies have demonstrated that the yeast Miro GTPase Gem1 influences mitochondrial morphology, inheritance, and mtDNA nucleoids in ways that parallel the effects of ERMES 86,90. Gem1 has been demonstrated to regulate the size and number of ERMES elements without affecting their assembly 91,92 (**Figure 1F**). A small GTPase gene, *ARF1*, genetically interacts with *GEM1* and *DNM1* to sustain mitochondria dynamics and function. Arf1/2 mutation caused abnormal Fzo1 clusters on mitochondria, increasing the ratio of cells with punctate mitochondria (**Figure 2H**). Similar mitochondrial network morphology defects are identified in cells with mutation of Gea1/2, the guanine nucleotide exchange factors of Arf1/2 91 (**Figure 2H**). Another small ER membrane GTPase, Sar1, has been implicated in regulating the size of ER-mitochondria contact sites through modulating membrane curvature and lipid exchange. The loss of Sar1 GTPase function leads to an abnormal morphology of aggregated mitochondria instead of the tubular form observed in wildtype yeast cells 93 (**Figure 2F**). In summary, ERMES plays a key role in stabilizing the membrane contacts of the ER and mitochondria, supporting inter-organellar material exchange, and regulating mitochondria dynamics.

## THE CYTOSKELETON SYSTEM ENGAGES IN THE REGULATION OF MITOCHONDRIA DYNAMICS

Since mitochondria cannot undergo *de novo* synthesis, their proper transport and segregation during asymmetric cell division are critical for inheritance 94. Mdm20 was initially discovered as a regulator of actin turnover, and the Δ*mdm20* strain possesses fewer actin cables and defective mitochondrial inheritance 95. The Arp2/3 complex was found to be required for mitochondrial transport on actin cables 96. Accumulating evidence has indicated that the Myo2-Mmr1-Ypt11 complex connects mitochondria as cargo and is transported along with actin cables to pass through the bud neck 97,98,99 (**Figure 1B**). Liza Pon’s group proposed a mitochondrial quality control mechanism during inheritance, termed retrograde actin cable ﬂow (RACF), in which depolarized mitochondria were transported in the retrograde direction along the actin cables to ensure healthy mitochondria in daughter cells 100. However, aggregated mitochondria are retained in mother cells with ERMES defects while the actin structure is not altered 101. These findings indicate the role of the actin cytoskeleton in selectively trafficking mitochondria during inheritance, thus maintaining the functional organelles in daughter cells.

The role of the actin cytoskeleton in mitochondrial transport has been intensively studied, yet its involvement in mitochondria dynamics has scarcely been discussed. The inhibition of actin polymerization by latrunculin A in yeast causes mitochondrial morphological alterations in a dose-dependent manner. When a vegetative cell is treated with a low dosage of approximately 0.5 μM latrunculin A, the mitochondria transition from a tubular to hyperfused morphology, whereas a higher dosage of latrunculin A induces mitochondrial fragmentation, which is dependent on Dnm1 and Fis1 25,102,103. We previously performed a yeast two-hybrid screening with Drp1 (a human homolog of Dnm1) as bait. One of the proteins that interacts with Drp1 is adenylyl cyclase-associated protein 2 (CAP2). The yeast homolog of CAP2 is Srv2 (Suppressor of RasVal19), also an actin-binding protein that regulates actin turnover 102,104. Our results demonstrated that Srv2 interacts with Dnm1 on mitochondria (**Figure 1B**). Deletion of *SRV2* causes hyperfused mitochondria (**Figure 2I**). This work characterized Srv2 as a pro-fission factor that modulates mitochondrial morphology and actin cables individually 102. So far, actin imaging in yeast has focused chiefly on actin cables since visualizing actin filaments remains challenging owing to the limitations of fluorescence microscopy. While higher-resolution techniques such as cryo-electron microscopy provide enhanced structural insights, it is unsuitable for real-time live imaging to study actin dynamics during mitochondrial fission. Studies in mammalian models have demonstrated that mitochondrial fission at the "midzone" area, rather than at tips, is coordinated together by both ER and actin 105. However, no evidence in yeast shows that both ER and actin participate in mitochondrial fission or are localized at fission sites. The roles of ER-actin interactions with mitochondria and actin filaments beyond cables in mitochondria dynamics await further investigation.

## THE PHYSIOLOGICAL ROLE OF MITOCHONDRIA DYNAMICS IN YEAST CELLS

Mitochondria are central hubs of signal transduction and metabolic pathways. The balance of mitochondria dynamics is affected by both cellular factors and environmental stress. Studies have indicated that distinct mitochondrial network morphologies are associated with specific physiological scenarios. This unique correlation indicates the role of mitochondria dynamics in each status change in cells.

Due to catabolic repression in yeast, energy is produced through glycolysis in a glucose-rich environment 106. During this phase, the balance of mitochondria dynamics drives a tubular network. When yeast cells are cultured in non-fermentable carbon sources like ethanol or glycerol, mitochondria form elongated/hyperfused and highly branched networks 107. The elongation phenotype enhances the formation of supercomplexes in the electron transport chain system and then increases the oxidative phosphorylation efficiency 3. Therefore, hyperfused mitochondria are often viewed as having an energetic phenotype upon changing the carbon source. However, shifting the dynamic balance toward fusion is not always the response to actively increasing oxidative phosphorylation to meet harsh conditions for survival. We recently reported that mitochondria undergo active fission under glucose depletion conditions after 48 hours of culture in the stationary phase. The ratio of cells with fragmented mitochondria is significantly reduced in the stationary* DNM1* deletion strain 108. An increased number of fragmented mitochondria may favor the selective degradation of disintegrated mitochondria to maintain overall mitochondrial quality. This irregular balance of mitochondria dynamics in long-term culture is like the changes in mitochondria dynamics in an acidic environment during tumorigenesis. Our findings demonstrated that maintaining mitochondria dynamics *per se* is critical for cells to encounter environmental challenges.

Since mitochondrial integrity is proposed to be a significant aging factor, mitochondria dynamics are being studied intensively in senescent yeast models. Damien Laporte *et al*. used mitochondrial morphology to distinguish the rejuvenation ability of chronological senescent yeast. After seven days of inoculation, the mitochondria transitioned into approximately 85% vesicular and 5% globular morphologies. They demonstrated the effects of nutrient refeeding, and only cells harboring vesicular mitochondria could re-enter proliferation. This finding indicates that the vesicular mitochondria type is a potential marker of quiescence and that globular mitochondria represent senescence 109. Vesicular mitochondria are often found in chronological senescent cells with the deletion of major factors involved in mitochondrial fission, Dnm1, Fis1, Mdv1, and Caf4. The phenotype implies that a minor mitochondrial fission mechanism needs to be characterized 109. In replicative senescent yeast cells, mitochondria tend to develop a fragmented morphology and produce additional reactive oxygen species 45,110. We previously demonstrated that mitochondrial fragmentation is associated with increased Dnm1 protein levels. Resveratrol can reduce the ratio of senescent cells with fragmented mitochondria and eliminate ROS 110. These findings are consistent with the work of ‪Christian Q. Scheckhuber *et al*., who reported that knockout of either *DNM1* or *FIS1* caused net-like mitochondria in replicative senescence. Suppressing mitochondrial fragmentation in senescent yeast results in decreased oxidative stress and an increased replicative lifespan 111. These reports demonstrated that mitochondria dynamics play a role in the physiological status of senescent cells.

There is increasing evidence correlating mitochondria dynamics and physiological adjustments in yeast cells. We recently demonstrated that fission-defective cells presented a lower desiccated survival rate 108. Qun Ren *et al*. used electron microscopy to reveal the mitochondrial structure and number of yeast cells from different growth phases after desiccation. Their results revealed that stationary cells maintain greater numbers of individual mitochondria than those in the log phase 112. These findings suggest that mitochondrial fragmentation is a protective process for desiccation tolerance. One study used alpha factor to induce G1 phase arrest in yeast cells and reported a fragmented mitochondrial network. This study revealed proteasomal degradation of the mitochondrial fusion factor Fzo1 in alpha-factor-arrested cells. The authors suggested that fragmented mitochondria were prepared for efficient fusion during zygote mating 113.

**Figure 3:**
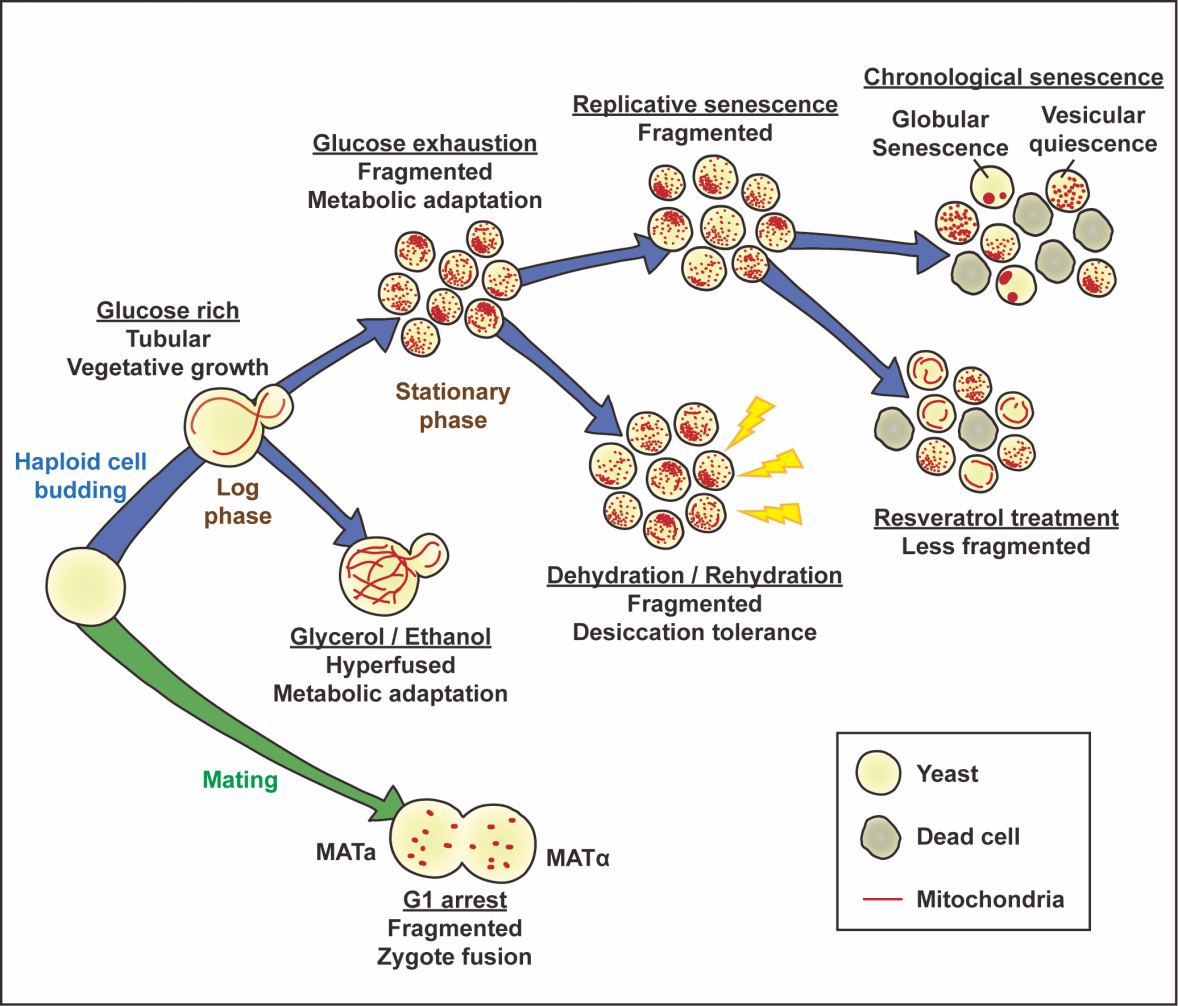
FIGURE 3: Physiological roles of mitochondria dynamics in yeast cells. The balance of mitochondria dynamics changes with physiological conditions in yeast cells. This figure depicts a mitochondrial network found under severe physiological conditions. Vegetative growing yeast cells present tubular mitochondria in the log phase. When cultured in a non-fermentable carbon source, mitochondrial morphology becomes hyperfused to support respiratory adaptation. Upon glucose exhaustion and entry into the stationary phase, mitochondria turn into fragments, which is the state associated with increased tolerance under desiccated conditions. The same fragmented phenotype is observed in replicative senescent cells, which can be restored by resveratrol treatment. During chronological aging, mitochondrial morphology changes into either a vesicular (fragment-like) or globular (large, bubble-like) form, representing quiescent cells (capable of re-entering the cell cycle) or senescent cells (irreversibly arrested), respectively. In addition, fragmented mitochondria are found during zygote fusion, facilitating the proper segregation of mitochondria. The regulatory pathways responsible for individual morphology under specific physiological conditions require intensive study to characterize.

The studies mentioned above are depicted in **Figure 3**. These examples emphasize the close correlation between mitochondria dynamics and physiological conditions.

## CONCLUSION AND PERSPECTIVE

Mitochondria dynamics are fascinating phenomena in cells. These processes also involve organellar communication and the sophisticated regulation of mitochondria, and previous studies have shed light on the regulatory mechanisms involved. However, the signaling pathways that translate environmental stimuli into a dynamic mitochondrial response and the corresponding adjustments in mitochondrial activity remain obscure. Clarifying these unanswered questions will benefit our pursuit of therapies for mitochondria-related disorders, such as neurodegenerative diseases and cancer. Although the yeast cell is the primary eukaryotic model, it contributes valuable findings that can be used to understand mitochondria dynamics. Unsurprisingly, the yeast model will continue to play a significant role in characterizing the role of mitochondria dynamics in cells.

## CONFLICT OF INTEREST

The authors declare no conflicts of interest.
